# Parental socioeconomic status and adolescent health in Japan

**DOI:** 10.1038/s41598-021-91715-0

**Published:** 2021-06-08

**Authors:** Shohei Okamoto

**Affiliations:** 1 Research Team for Social Participation and Community Health, Tokyo Metropolitan Institute of Gerontology, 35-2 Sakae-cho, Itabashi-ku, Tokyo, Japan; 2 Institute for Global Health Policy Research, National Centre for Global Health and Medicine, 1-21-1 Toyama, Shinjuku-ku, Tokyo, Japan

**Keywords:** Epidemiology, Outcomes research, Paediatric research, Health policy, Epidemiology

## Abstract

There is no consensus on which parental socioeconomic indicators should be used to define adolescents’ socioeconomic status (SES). Utilising the data for 3154 parent-adolescent pairs obtained from the sample of the *Survey of Lifestyle Value of Parents and Children 2011* conducted by the Cabinet Office in Japan, the associations between adolescent’s subjective economic status, parental SES (i.e. education, occupation, and household income), and child health-related outcomes (i.e. self-rated health, dietary and oral health behaviours) were analysed using multilevel mixed-effects ordered logistic regression to investigate heterogeneity in these relationships across SES indicators and health outcome measures. Results demonstrated that income was the strongest predictor of adolescent health outcomes, suggesting that adolescents in the middle- or high-income groups tended to report better health status compared to the low-income group, have a higher frequency of having breakfast, and more likely to regularly brush their teeth by 24% (OR 1.24, 95% CI [1.06–1.46]) to 66% (OR 1.66, 95% CI [1.30–2.12]). Parental education was also related to child health-related behaviours, with higher levels of habitual healthy behaviours being observed in the middle- and high-education groups than in the low-education group by 15% (OR 1.15, 95% CI [1.01–1.32]) to 63% (OR 1.63, 95% CI [1.31–2.03]). Future studies regarding health disparities among children/adolescents should carefully choose an SES indicator, taking multiple pathways between each SES indicator and health/health behaviours into consideration.

## Introduction

### Socioeconomic status and health in the context of a life-course approach

Socioeconomic status (SES) and health status during childhood are meaningful determinants of health in later life. This relationship has received considerable academic attention (for reviews, see, for example, Galobardes et al.^1^). While health is an important means of determining SES (i.e. health selection), the reverse pathway, or the effects of socioeconomic environments on health, also exists (i.e. social causation)^2–4^. Therefore, disparities in health and socioeconomic status during childhood are important health and social issues, which should be addressed by appropriate policies.

There are many theoretical explanations for the relationship between SES in early life and general health in later life, including *the critical period (or latent effects) model, accumulation of risks*, and the *chains of risk*^5–7^. The *critical period model* suggests that disadvantageous circumstances occurring in critical periods of life, such as when vital biological developments take place in utero, have lasting or latent impacts on later health. Alternatively, the *accumulation of risks* theory proposes that ill-health is induced by exposure to disadvantages throughout life, while the *chains of risk* model contends that a risk that a negative factor can trigger the subsequent recurrence of this same negative factor. Furthermore, low SES during childhood is associated with poor behavioural choices, such as cigarette smoking, which increases the risk of future disease^8^. Previous studies have reported that health disparities among different socioeconomic groups among adults can be partly explained by the poor health-behaviour choices^9,10^, which may be oriented from childhood experiences.

Adverse socioeconomic circumstances also restrict an individual’s access to other means of investing in human capital (e.g. education). Thus, these conditions are likely to persist or worsen even after they become adults for children with adverse health and low SES^2–4^. In fact, empirical research has found that parental poverty negatively influences their children’s health and educational attainment, such that the children show worsened health and SES in adulthood as a result of bad health and low labour productivity^11,12^. Moreover, health disparities already exist during childhood, and the gradients are larger among older children^13–16^. Thus, an investigation of the impact of childhood socioeconomic disadvantages on their health status is critical to increase the understanding of their relationship, which can help identify methods of improving related social policy.

### Parental SES and child health

While previous studies have assessed the association of SES indicators and child health, there is no consensus regarding how to measure children’s/adolescents’ SES. SES denotes the relative social position of an individual or family, which influences their access to financial, social, cultural, and human capital resources, and is often measured by the ‘Big 3 Variables’ (i.e. education, occupation, and income)^17–19^. For children and adolescents who are students, subjective social class (or economic well-being) or a Big 3 Variable of parents is frequently used to measure their SES^17,20–25^. Of these three commonly used SES indicators, income may reflect one’s level of SES better than the other measures and predict necessary resources to maintain good health^19^, as income is mainly determined by occupation, which is influenced by educational attainment.

Meanwhile, different parental SES indicators, even the most commonly used variables (i.e. education, occupation, and income), could affect health through unique pathways. These socioeconomic indicators are likely to have intellectual, material, and psychological effects on health and health behaviours^19,25–29^. While all Big 3 Variables, as well as subjective economic status could affect the psychological health of children through the chronic stress and social stigma associated with adverse socioeconomic circumstances, educational attainment has been linked to knowledge and non-financial aspects affecting health behavioural choices^30^. Therefore, parental educational attainment and, thus, their health literacy may affect the formation of healthy lifestyles in their children. Occupation reflects one’s social status and job-related material resources, and parental occupation can affect children’s health through the material conditions and psychological factors derived from occupational prestige and job conditions^25,31^. Household income, primarily based on parental earnings, affects access to materials and services along with the purchasing power to maintain good health.

Although each SES indicator may have unique underlying mechanisms related to child health, children and adolescents may not provide accurate information regarding parental SES, such as parental occupation and income^25,32,33^. Therefore, some studies have elected to use adolescents’ subjective social status to measure SES^21,24^, shifting the primary focus to the psychological effects of SES on children.

Given the existing research, the present study aimed to investigate, using a sample of Japanese adults and adolescents, the relationship of the parental Big 3 SES Variables (i.e. education, occupation, and income) and economic status assessed by adolescents with adolescents’ health-related outcomes. A notable strength of this study is that adolescents’ surveys were linked to the concurrent surveys of their parents, facilitating more accurate measurements of parental SES than relying on adolescents’ self-reported SES, which frequently generates non-responses or inaccurate reports for parental SES^25,32,33^. Further, this design can determine how each parental SES indicator is associated with child health-related outcomes.

## Method

### Study design and sample

Data were derived from the Japanese Cabinet Office’s *Survey of Lifestyle Value of Parents and Children 2011*. The survey aimed to determine the level of support required to relieve children from certain adverse circumstances by investigating parents’ and children’s values and attitudes regarding various aspects of life (e.g. education and occupation). The survey was administered by leaving method between October and November 2011 to ninth-grade students (14- or 15-year-olds) and their guardians. The sample was recruited from 240 areas across Japan and was extracted using a stratified two-stage random sampling method. Stratification was first performed at the prefectural level and then by the population and city scale. The response rate was 79.8% (n = 3192) for the adolescents and 79.9% (n = 3197) for the guardians, comprising responses from 3178 pairs of adolescents and their guardians. In instances where the guardians’ survey was not answered by a parent (e.g. completed by an older brother or sister, grandparent, or parent-in-law), the responses were excluded from the analysis (n = 24) because the information such individuals could provide regarding parental SES was limited. Ethical approval was not required as this study was based on the secondary analysis of publicly available data.

### Health and SES variables

From the adolescents’ survey, the included variables were self-rated health, dietary and oral health behaviours, and subjective economic status. Self-rated health was measured using a five-point rating scale on which children’s perceived health status was rated from ‘bad’ to ‘good’. Self-rated health is a validated scale of predicting future mortality and morbidity^34^, and is also validated for adolescents^35^. The survey included questions regarding dietary and oral hygiene behaviours. To measure dietary behaviour, the frequency with which one skipped breakfast was rated on a four-point scale (‘do not have breakfast at all’, ‘rarely have breakfast’, ‘sometimes skip breakfast’, and ‘have breakfast every day’). Skipping breakfast has been associated with poor health and other negative health behaviours in adolescents (e.g. smoking, infrequent exercise, frequent alcohol use, and high body mass index)^36,37^. Oral health behaviour was measured by the frequency of toothbrushing rated on a four-point scale (’rarely’, ‘sometimes’, ‘once a day’, and ‘twice a day or more’). Adolescent oral health behaviour is important since tooth decay is the most common chronic disease^38^. As a SES indicator previously used^21,24^, adolescents self-assessed their economic status, ranging from ‘very poor’ to ‘very wealthy’.

From the survey of parents, the parental SES indicators (i.e. income, education, and occupation) were examined. Income was assessed by the gross household income in the past year, which was transformed into equalised household income by dividing it by the square root of the number of household members. The equalised income was then classified into tertiles, corresponding to low-, middle-, and high-income groups. Education was also divided into three groups: ‘high-school graduate or lower’, ‘junior college or vocational school graduate’, and ‘university graduate or higher’. Finally, employment status was used for occupation, as the survey did not investigate job classification. Employment status categories included ‘not employed’, ‘full-time worker or executive’, ‘self-employed or other types of job’, and ‘non-regular worker’. As the information regarding education and occupation were obtained for both fathers and mothers when available, adolescent SES was based on the higher status for the father’s or mother’s education and occupation.

### Statistical analyses

Based on previous research^21,24^, the associations between adolescents’ subjective economic status and the different objective parental SES indicators was analysed. Further, the potential heterogeneity in the associations between different SES indicators and adolescent health outcomes was examined. Since education, occupation, and income can be intercorrelated, their multicollinearity can influence the associations between SES indicators and adolescent health-related outcomes when including all parental SES variables. Therefore, after analysing the associations between income, education, and occupation, a two-step procedure was adopted following previous research^29^. First, the associations between each parental SES indicator and adolescent health-related outcomes were analysed after controlling for being in a single-parent household, father’s and mother’s age, and scale of the residential area. Second, the associations between each parental SES indicator and the adolescents’ health-related outcomes were assessed, controlling for the other two SES indicators and the same control variables as the first step. This second step was adopted to analyse the relationships between the adolescents’ self-assessed economic status and health-related outcomes.

Given the clustered structure of the data, multilevel mixed-effects ordered logistic regression was used, fitting a three-level model incorporating school type (public, national, private, special-needs schools, and other types) nested within residential areas (Hokkaido, Tohoku, Kanto, Hokuriku, Tozan, Tokai, Kinki, Chugoku, Shikoku, Kitakyushu, and Minamikyushu). Clustering at the parent–child level was not addressed since the current analyses comprised different variables from the two respondents (e.g. the child’s health and parent’s SES); thus, the data were not regarded as dyadic data^39^.

Multiple imputations were used to address the missing data with the assumption of missing data at random, using variables from both the children and parent surveys (including children’s school performance, living conditions, parent–child relationships, parents’ attitudes toward education, and means of discipline for the child). The final sample size included 3,154 pairs of adolescents and parents. All analyses were conducted using Stata software, version 16.1 (StataCorp LLC, College Station, United States of America), with ORs and 95% CIs being estimated based on cluster-robust standard errors, which allowed for intragroup correlations to address heteroskedasticity across individuals.

## Results

Table 1 shows the descriptive statistics for the variables concerning children’s status. About half (47.5%) of the respondents were female students, and more than half rated their economic status as fair (57.0%). The majority of children assessed their health as good (48.1%), had breakfast every day (83.4%), and brushed their teeth twice a day or more (67.3%).

**Table 1:** Descriptive statistics for children.

Variable	Proportion (%)
Female	47.5
**Subjective economic status**	
Very poor	5.3
Somewhat poor	17.8
Fair	57.0
Somewhat wealthy	14.8
Very wealthy	5.1
**Self-rated health**	
Bad	0.9
Somewhat bad	4.4
Fair	23.5
Somewhat good	23.0
Good	48.1
**Breakfast**	
Not at all	1.1
Hardly	3.8
Sometimes skip	11.7
Have everyday	83.4
**Tooth brushing**	
Hardly	1.0
Sometimes	5.3
Once a day	26.5
Twice a day or more	67.3
**School type**	
Public	87.0
National	1.5
Private	10.2
Special-needs	0.5
Other types	0.7

Table 2 presents the descriptive statistics for the parental and household characteristics. The average ages of the father and mother were 46.8 (standard deviation [SD]: 5.5) and 44.2 (SD: 4.4) years old, respectively. The average equalised income was approximately 3 million JPY (≈ 24,000 EUR). University graduates or higher was the most common (42.6%), and more than two-thirds (76.7%) worked as a full-time worker or executive.

**Table 2:** Descriptive statistics for parents and households.

Variables	Mean (SD) or proportion
Age of father	46.8 (5.5)
Age of mother	44.2 (4.4)
**Education**	
High school or lower	30.4%
Junior college/vocational school	27.0%
University or higher	42.6%
**Employment status**	
Not in employment	3.7%
Full-time or executives	76.7%
Self-employment	9.7%
Non-regular worker	9.9%
Equalised household income (1 million JPY)	300.0 (155.7)
**Scale of residential area**	
Designated cities by government ordinance	22.0%
Population: 200,000 or more	24.8%
Population: 100,000 or more	17.3%
Population: less than 100,000	24.7%
Town or village	11.1%
**Residential area**	
Hokkaido	4.3%
Tohoku	7.9%
Kanto	28.2%
Hokuriku	5.1%
Tozan	5.1%
Tokai	10.9%
Kinki	15.5%
Chugoku	6.7%
Shikoku	3.5%
Kitakyushu	7.1%
Minamikyushu	5.7%

Table 3 shows the associations between the subjective economic status of the children and the objective parental SES measures, estimated by the multilevel ordered logistic regression. Children’s assessment of their economic status was positively associated with parental SES indicators. Children rated their economic status significantly higher when their parents’ educational attainment (Junior College or Vocational School: OR 2.22, 95% CI [1.86–2.64]; University or Higher: OR 5.27, 95% CI [4.25–6.54]) and income were higher (Middle Income: OR 2.22; 95% CI [1.86–2.64]; High Income: OR 5.27; 95% CI [4.25–6.54]). Similarly, children with self-employed parents (OR 1.68, 95% CI [1.06–2.67]) or parents in a full-time or executive job (OR 1.58, 95% CI [1.11–2.25]) were likely to report their economic status was higher than those who were not in paid work.

**Table 3﻿:** The association between economics status assessed by children and objective parental SES indicators.

Subjective economic status (1: very poor–5: very wealthy)	
**Parent education: high school or lower (reference)**	
Junior college or vocational school	2.22** (1.86–2.64)
University or higher	5.27** (4.25–6.54)
**Parent employment status: not employed (reference)**	
Full-time worker or executive	1.58* (1.11–2.25)
Self-employed	1.68* (1.06–2.67)
Non-regular worker	1.14 (0.77–1.71)
**Household income: low (reference)**	
Middle	2.22** (1.86–2.64)
High	5.27** (4.25–6.54)
Observations	3154
Number of imputations	20

These results were estimates by multilevel ordered logistic regression, controlling for sex of a child, parental ages, being in a single-parent household, and the scale of a residential area and fitting a three-level model incorporating school type nested within residential areas.

Values are odds ratios and 95% confidence intervals based on robust standard errors in parenthesis with *p < 0.05, **p < 0.01.

Table 4 presents the bivariate associations between the pairs for parental education, parental occupation, and household income. Educational attainment of parents who had a full-time or executive job was found to be higher (OR 2.68, 95% CI [1.59–4.54]). Also, higher household income was associated with having a full-time or executive job (OR 5.91, 95% CI [3.70–9.44]) and having a higher level of education (Junior College or Vocational School: OR 1.82, 95% CI [1.45–2.27]; University or Higher: OR 4.73, 95% CI [3.76–5.95]). Therefore, these three SES indicators were associated with each other.

**Table 4:** Associations between the pairs among parental education, parental occupation, and household income.

	Education	Income
**Education**		
Low (Ref.)		
Middle		1.82** (1.45–2.27)
High		4.73** (3.76–5.95)
**Occupation**		
Not employed (Ref.)		
Full-time or executive	2.68** (1.59–4.54)	5.91** (3.70–9.44)
Self-employed	1.60 (0.91–2.82)	1.46 (0.91–2.34)
Non-regular worker	1.10 (0.57–2.09)	0.74 (0.46–1.18)
Number of imputations	20	
N	3154	

These results were estimates by multilevel ordered logistic regression, controlling for parental ages, being in a single-parent household, and the scale of a residential area and fitting a three-level model incorporating school type nested within residential areas.

Values are odds ratios and 95% confidence intervals based on robust standard errors in parenthesis with *p < 0.05, **p < 0.01. The bivariate associations between the pairs among parental education, parental occupation, and household income with aforementioned controls were analysed.

Table 5 shows the relationships between parental SES and adolescent health-related outcomes. In the first step (Model 1), income was robustly associated with all child health outcomes. Adolescents with parents in middle and high-income groups were more likely than those of parents in the low-income category to report better health (Middle Income: OR 1.26, 95% CI [1.09–1.47]; High Income: OR 1.34, 95% CI [1.12–1.60]), have breakfast more frequently (Middle Income: OR 1.46, 95% CI [1.20–1.78]; High Income: OR 1.97, 95% CI [1.59–2.44]), and brush their teeth more routinely (Middle Income: OR 1.21, 95% CI [1.03–1.41] ]; High Income: OR 1.58, 95% CI [1.30–1.93]). Parental educational attainment was positively associated with health behaviours, but not adolescent self-rated health. Children with parents having Junior College or Vocational School and University Graduate or Higher compared to low were more likely to have breakfast regularly (Middle Education: OR 1.26, 95% CI [1.04–1.53]; High Education: OR 1.63, 95% CI [1.31–2.03]) and brush their teeth routinely (Middle Education: OR 1.15, 95% CI [1.01–1.32]; High Education: OR 1.57, 95% CI [1.25–1.98]). However, there were no differences in child health-related outcomes depending on the parental occupation.

**Table 5:** Associations between parental SES and child health behaviours/health outcomes.

	SRH	Breakfast	Toothbrushing
**Model 1**			
Education			
Low (Ref.)			
Middle	1.07 (0.95–1.22)	1.26* (1.04–1.53)	1.15*(1.01–1.32)
High	1.10 (0.93–1.30)	1.63** (1.31–2.03)	1.57** (1.25–1.98)
Occupation			
Not employed (Ref.)			
Full-time or executive	1.32 (0.87–2.00)	1.52 (0.92–2.50)	1.44 (0.97–2.14)
Self-employed	1.18 (0.76–1.84)	1.04 (0.56–1.94)	1.15 (0.79–1.65)
Non-regular worker	1.14 (0.79–1.65)	1.08 (0.79–1.48)	1.05 (0.69–1.61)
Income			
Low (Ref.)			
Middle	1.26** (1.09–1.47)	1.46** (1.20–1.78)	1.21* (1.03–1.41)
High	1.34** (1.12–1.60)	1.97** (1.59–2.44)	1.58** (1.30–1.93)
**Model 2**			
Education			
Low (Ref.)			
Middle	1.03 (0.90–1.17)	1.17# (0.98–1.40)	1.11 (0.96–1.28)
High	1.00 (0.85–1.18)	1.37** (1.10–1.71)	1.41** (1.11–1.79)
Occupation			
Not employed (Ref.)			
Full-time or executive	1.20 (0.79–1.81)	1.24 (0.73–2.10)	1.26 (0.82–1.92)
Self-employed	1.16 (0.73–1.83)	0.99 (0.52–1.90)	1.11 (0.78–1.59)
Non-regular worker	1.14 (0.78–1.67)	1.09 (0.78–1.51)	1.06 (0.67–1.67)
Income			
Low (Ref.)			
Middle	1.24** (1.06–1.46)	1.33** (1.08–1.64)	1.10 (0.92–1.32)
High	1.32** (1.12–1.56)	1.66** (1.30–2.12)	1.33** (1.10–1.61)
Number of imputations	20		
N	3154		

These results were estimates by multilevel ordered logistic regression, controlling for sex of a child, parental ages, being in a single-parent household, and the scale of a residential area and fitting a three-level model incorporating school type nested within residential areas.

Values are odds ratios and 95% confidence intervals based on robust standard errors in parenthesis with *p < 0.05, **p < 0.01. The bivariate associations of each SES indicator and child health outcomes with aforementioned controls were analysed in the model 1, while the model 2 included all three SES indicators.

In the second step (Model 2), the association between each parental SES indicator with adolescent health outcomes was analysed after controlling for the other two SES indicators. While the associations were attenuated, some of the relationships observed in the Model 1 remain unchanged. All health outcomes of children, particularly among the high-income group, were better than those in the low-income group: (Self-Reported Health: OR 1.32, 95% CI [1.12–1.56]; Eating Breakfast: OR 1.66, 95% CI [1.30–2.12]; Toothbrushing Habits: OR 1.33, 95% CI [1.10, 1.61]). No occupational differences were observed.

As Table 6 indicates, parental education was associated with child health behaviours. Potential heterogeneity by the parent’s sex was examined by analysing the associations between fathers’/mothers’ education and adolescent health behaviours, restricting the analysis to non-single-parent households to compare across members of the same subgroup. Two models were considered: models including educational attainment for (1) each parent separately and (2) both parents simultaneously, controlling for child sex, parental ages, and the scale of a residential area. Although mechanisms underlying the linkage may differ between fathers and mothers, which is beyond the scope of this study, both parents’ educational attainments were positively associated with child health behaviours.

**Table 6:** Associations between fathers’/mothers’ education and child health behaviours.

	Education	Breakfast	Toothbrushing
Model 1	**Father**		
	Low (Ref.)		
	Middle	1.51* (1.06–2.15)	1.15*(1.01–1.32)
	High	1.68** (1.38–2.04)	1.57** (1.25–1.98)
	**Mother**		
	Low (Ref.)		
	Middle	1.19* (1.01–1.40)	1.11 (0.96–1.28)
	High	1.80** (1.45–2.23)	1.41** (1.11–1.79)
Model 2	**Father**		
	Low (Ref.)		
	Middle	1.48* (1.02–2.15)	0.96 (0.77–1.20)
	High	1.54** (1.25–1.89)	1.32* (1.02–1.70)
	**Mother**		
	Low (Ref.)		
	Middle	1.05 (0.90–1.22)	1.26** (1.09–1.45)
	High	1.43** (1.16–1.77)	1.47** (1.11–1.94)
Number of imputations		20	
N		2681	

These results were estimates by multilevel ordered logistic regression, controlling for sex of a child, parental ages, and the scale of a residential area and fitting a three-level model incorporating school type nested within residential areas. The sample was restricted to non-single-parent households. Educational attainment for each parent was included in the Model 1, while both parents’ educational attainments were included in the Model 2.

Values are odds ratios and 95% confidence intervals based on robust standard errors in parenthesis with *p < 0.05, **p < 0.01.

Table 7 presents the association between adolescents’ subjective economic status and their health-related outcomes. The associations were analysed without including objective SES indicators (i.e. parental education, parental occupation, and household income) in Model 1, but with all three SES indicators in Model 2. Compared to children reporting very poor economic status, those who reported being in the higher economic status groups (Fair–Very Wealthy) had better outcomes for all health-related outcomes, with ORs ranging from 1.47 (95% CI [1.01–2.12]) to 2.82 (95% CI [1.66–4.79]). Even after adjusting SES indicators, this pattern was still observed for children whose subjective economics status was reported as fair to very wealthy, had ORs ranging from 1.48 (95% CI [1.15–1.91]) to 2.25 (95% CI [1.31–3.86]).

**Table 7:** Associations between subjective economic status and child health behaviours/health outcomes.

	SRH	Breakfast	Tooth brushing
**Model 1**			
Subjective economic status			
Very poor (Ref.)			
Somewhat poor	0.94 (0.66–1.34)	0.99 (0.61–1.60)	1.15 (0.95–1.40)
Fair	1.47* (1.01–2.12)	1.99** (1.31–3.03)	1.63** (1.30–2.05)
Somewhat wealthy	1.74** (1.27–2.38)	2.82** (1.66–4.79)	2.05** (1.58–2.65)
Very wealthy	2.07** (1.35–3.19)	2.69** (1.78–4.06)	1.98* (1.16–3.39)
**Model 2**			
Subjective economic status			
Very poor (Ref.)			
Somewhat poor	0.91 (0.64–1.31)	0.93 (0.57–1.50)	1.10 (0.91–1.34)
Fair	1.40 (0.98–2.00)	1.75** (1.14–2.67)	1.48** (1.15–1.91)
Somewhat wealthy	1.64** (1.22–2.19)	2.25** (1.31–3.86)	1.70** (1.30–2.24)
Very wealthy	1.95** (1.33–2.85)	2.15** (1.33–3.46)	1.65 (0.91–3.00)
Number of imputations	20		
N	3154		

These results were estimates by multilevel ordered logistic regression, controlling for sex of a child, parental ages, being in a single-parent household, and the scale of a residential area and fitting a three-level model incorporating school type nested within residential areas.

Values are odds ratios and 95% confidence intervals based on robust standard errors in parenthesis with *p < 0.05, **p < 0.01. The bivariate associations of economic status assessed by children and child health outcomes with aforementioned controls were analysed in the model 1, while the model 2 adjusted for all three SES indicators.

## Discussion

This study investigated the association between different parental SES indicators (i.e. education, occupation, and household income) and health-related outcomes of adolescents, using data from both adolescents and their parents, and linking adolescent-reported health-related outcomes to parental-reported SES indicators. There were three notable major findings. First, adolescent-reported economic status was associated with parent-reported education, occupation, and household income, suggesting that adolescents’ subjective economic status may be used as an SES indicator for adolescents. Second, heterogeneous associations of parental SES indicators were found with child health-related outcomes. While household income was robustly associated with all health-related outcomes (i.e. self-reported health, the frequency of having breakfast, and toothbrushing habits), parental education alone was associated with adolescent-reported health behaviours. However, there was not a significant association between parental occupation and adolescent health-related outcomes. Although these three indicators are associated with each other, and multicollinearity is a potential concern, these patterns of association were unchanged even after simultaneously controlling for the other SES indicators. Third, adolescents’ self-assessed economic status was positively associated with their self-reported health outcomes, even adjusting for the parental objective SES indicators.

Only few studies have focused on comparing different SES measures across various child health-related outcomes. Parental occupational status using the International Standard Classification of Occupations was robust measures of SES for adolescents^25^, and SES indicators related to adolescents’ own education and perception of relative family SES, rather than parental objective SES, were strong predictors^40^. Further, previous research using data from adults provide some insights. Studies suggest that income was determined as the strongest predictor of health^28^, heterogeneity was observed depending on types of health outcomes^29^, and each SES indicator (i.e. education, occupation, and income) indirectly contributed to health gradients via mediating variables^19^, interpreting their findings as health consequences throughout multiple pathways of different SES indicators. Together with the findings of this study, household income appears to be the most robust SES indicator among the Big 3 Variables, while parental education, linked to their health literacy, can be useful in predicting children’s health behaviours^41^.

Consistent with the current findings, a meta-analysis indicated that adolescents’ subjective economic status was associated with their health outcomes, particularly mental health, even after adjusting for objective SES indicators^24^. These findings suggest that using only specific objective SES indicators may insufficiently capture potential influences from one’s SES, such as the psychological pathway. Although addressing this causal relationship is beyond the scope of this study, reverse causality between children’s subjective economic status and child health-related outcomes is of concern—adolescents whose health, including mental health, is worse (e.g. those who are depressed) may be more likely to report economic difficulty than healthy individuals. Further research is needed to verify the directionality of this association.

These findings suggest important policy implications for improving children’s health and mitigating health disparities. As disparities in health behaviours and self-rated health among adolescents across different levels of SES exist, addressing inequality in parental SES and social determinants of health that can impact child health-related outcomes is essential. From a life-course perspective, the effects of adverse socioeconomic conditions and poor childhood health could result in chronic, deteriorating health conditions and restrict economic opportunities in later life. Therefore, providing adequate cash and in-kind benefits for socioeconomically disadvantaged households with children is particularly important to reduce the intergenerational transmission of poverty and ill health. Moreover, the necessary supports may vary due to the potential heterogeneity in the mechanisms leading to the relationships between SES indicators and health and the desired health-related outcome targeted by policymakers. Financial and non-financial supports, such as cash benefits and vouchers, can improve children’s health by enhancing their access to materials, services, and purchasing power needed to maintain good health. Conversely, enhancing parents’ and children’s health literacy through education can help ameliorate children’s future health disparities by influencing their health behaviours and choices.

The unique strengths of this study include its analytic methods and sample. The design allowed for the comparisons of different SES variables in relation to various adolescent health-related outcomes by using the surveys completed concurrently by adolescents and their parents, increasing the accuracy in measuring parental SES. However, there are several limitations. First, responses were obtained through self-report questionnaires. Self-reported measures can contain reporting errors; thus, objective measures of health status, health behaviours, and socioeconomic information would help make the analyses more precise, specific, and informative. An association existed between adolescent-reported subjective economic well- and their health-related outcomes. However, as described earlier, this result must be interpreted with caution because simultaneous bias can arise, and a further assessment of the validity of the scale would be required.

Further, there were no significant occupational differences in child health-related outcomes potentially because employment status, the only available indicator for parental occupational status rather than occupational class, was used. Thus, the inclusion of job-specific factors (e.g. occupational prestige and work hours) in this study was not possible.

Third, this study used a cross-sectional design, meaning it was not possible to detect trajectories in the relationships between socioeconomic disadvantages and health-related outcomes or control for time-invariant unobserved heterogeneity. In the context of the Japanese culture, investigating the long-term trajectory of health disparities from early childhood into adulthood may be necessary. Health (behavioural) disparities might increase after compulsory education since many students’ health behaviours benefit from access to school lunches in Japan (93.5% implementation rate at elementary and junior high schools in 2018)^42^, and physical activity and exercise are provided through classes and club activities.

In conclusion, this study found that household income was the strongest predictor of adolescent self-rated health and health behaviours while parental education was related to child health-related behaviours. Although further research and replications are needed, adolescent-assessed economic status can be useful to predict child health-related outcomes. Future research on health disparities among children should carefully choose an SES indicator, considering the multiple potential pathways between each SES indicator and health behaviours.
